# Navigating sustainable practice: environmental awareness and climate change as mediators of green competence of nurses

**DOI:** 10.1186/s12912-025-03313-4

**Published:** 2025-06-17

**Authors:** Nora Mahdy Attia, Alaa Eldin Moustafa Hamed, Mohamed Adel Abd Elhafeez Elbakry, Abeer Moustafa Barakat, Heba Sobhy Mohamed

**Affiliations:** 1 Assistant Professor of Nursing Administration, Faculty of Nursing, Zagazig University, Zagazig, Egypt; 2 Lecturer of Psychiatric and Mental Health Nursing, Faculty of Nursing, Helwan University, Cairo, Egypt; 3 Lecturer of Nursing Administration, Faculty of Nursing, Zagazig University, Zagazig, Egypt; 4 Lecturer of Maternal and Newborn Health Nursing, Faculty of Nursing, Helwan University, Cairo, Egypt; 5 Lecturer of Nursing Administration, Faculty of Nursing, Zagazig University, Zagazig, Egypt

**Keywords:** Green competence, Nurses’ sustainable practice, Environmental awareness and climate change

## Abstract

**Background:**

The sustainability of biological, social, and economic systems is essential to safeguarding our collective future and maintaining a balanced relationship between humans and the natural environment. Addressing environmental concerns requires the active involvement of all societal sectors, integrating sustainability awareness into everyday practices and business processes through optimal technology use. This study aims to examine the mediating role of climate change and environmental awareness in the relationship between sustainable practices and green competence among nurses.

**Subject and Methods:**

A random sample of 230 nurses was selected from Al-Ahrar Teaching Hospital in Zagazig, Egypt. A descriptive correlational design was used. Five validated instruments were used to assess sociodemographic characteristics, green competence, nurses’ perceptions of climate change, environmental awareness, and sustainable development behaviors.

**Results:**

The results of the current study show that green competence was significantly and positively correlated to nurses’ sustainable practice, environmental awareness and climate change.

**Conclusion:**

Environmental awareness and climate change were mediated of relationship between sustainable practice and green competence.

**Implications for Nursing Management:**

The findings of this study have significant implications for nursing management and the broader healthcare sector. By elucidating the relationships among green competence, nurses’ sustainable practices, environmental awareness, and climate change, this research offers actionable insights for healthcare leaders. Nursing managers can enhance workplace safety and environmental responsibility by supporting green management systems and implementing ongoing green management programs to improve nurses’ knowledge and attitudes toward sustainability. Furthermore, healthcare facilities should be encouraged to adopt environmental awareness and green management practices to address climate change and foster sustainable practices across the healthcare workforce.

**Clinical trial number:**

Not applicable.

## Background

The need for sustainable practices has become progressively urgent due to global environmental changes, especially in the healthcare sector. Organizations that successfully implement green strategies can achieve a lasting competitive advantage by reducing adverse environmental impacts and enhancing their green performance [1, 2]. The term *sustainable practice* refers to a comprehensive system of methods aimed at managing, restoring, and enhancing human health with an environmental foundation [3]. It encompasses three core aspects social, economic and environmental performance and operates in harmony with both the human body and the surrounding environment, aiming to balance impacts across all key components of the healthcare system [4, 5].

The long-term objective of maintaining a safe environment for present and future generations is conceptually connected to sustainable practice in nursing. Given the moral imperative to adopt a health-in-all-policies approach, the healthcare industry must immediately become more sustainable and ecologically conscious [6]. According to Danirmala and Prajogo (2022), green competency refers to the skills needed to adapt products, services, and processes to meet climate change challenges and comply with relevant legal and environmental requirements. This competency is essential across all workforce levels and sectors [7, 8]. As described by Anwar and colleagues (2020), *green competence* is the capacity to engage constructively and with heightened enthusiasm in one’s immediate environment. Indicators of green competence building include green knowledge, skills, abilities, attitudes, conduct, and awareness [9].

Additionally, the green competency variable can be classified into three main areas: green creativity, green expertise, and green task motivation [10]. Environmental awareness refers to an individual’s examination of, or response to, evidence, attitudes, or behaviors that impact the natural habitat [11]. Environmentally conscious individuals are more likely to recognize the risks of environmental contamination, collaborate to mitigate or eliminate these issues, and actively seek solutions [12, 13].

Climate change and environmental degradation pose escalating threats to human health and healthcare systems, with both direct and indirect impacts on physical and mental health. Direct effects include increased rates of mental health disorders—such as mood disturbances, irritability, anxiety, physical weakness, hypertension, headaches, hyperalgesia, autonomic symptoms, and insomnia. Indirect effects encompass conditions like chronic obstructive pulmonary disease (COPD), cancer, heart attack, stroke, and vector-borne diseases (e.g., dengue, malaria, Zika). Additionally, climate change contributes to sea level rise, floods, wildfires, storms, and hurricanes, and can result in health complications such as heart failure, malnutrition, and dehydration [14].

Furthermore, WHO projects nearly 250,000 additional deaths between 2030 and 2050 due to health issues exacerbated by climate change, including malnutrition, malaria, and diarrhea [15]. It is fundamentally unfair that those who contribute the least to climate change are often the most affected. This disparity impacts human rights as well as social and environmental factors, leading to forced migrations, social conflicts, and widening disparities among vulnerable groups such as Aboriginal populations, the elderly, migrants, individuals with chronic illnesses, and low-income communities [16].

Today, the negative effects of climate and environmental problems on health are gradually increasing [17]. Nurses, who play a pivotal role in illness prevention and health promotion across various settings, are key actors in addressing climate and environmental challenges [18]. This study explores how climate change and environmental awareness mediate the relationship between sustainable practices and the green competence of nurses.

To the best of the researchers’ knowledge, studies in the healthcare sector are limited. Wang and colleagues, (2022) found that green competency is a significant positive predictor of sustainability [19]. Similarly, Al Issa and colleagues, (2023) reported a positive association between green performance and sustainability [2]. Incesu and Altiner, (2023) asserted that a positive correlation exists between awareness of global climate change and environmental literacy, noting that participation in environmental discussions increases climate change awareness [20]. Liu and Cao, (2024) further reported that higher environmental awareness among employees leads to enhanced promotion of sustainable development performance [21].

This study is theoretically grounded in the Natural Resource-Based View (NRBV) of the firm, an extension of the Resource-Based View (RBV) developed by Hart (1995) [22], which emphasizes the role of environmental capabilities such as green competencies in achieving sustainable outcomes. The NRBV posits that firms, or by extension healthcare institutions, can gain competitive advantage by developing capabilities that support environmental sustainability. Applying this lens to the nursing context, green competence represents a strategic capability that enables nurses to manage environmental issues effectively and engage in sustainable practices. Environmental awareness and climate change perception serve as dynamic capabilities that translate these competencies into practice, thereby justifying the hypothesized mediation pathways.

While previous research has established positive associations among green competence, environmental awareness, and sustainability, there remains a paucity of studies examining the mediating effects of environmental awareness and climate change perception on the relationship between green competence and sustainable nursing practices particularly within the healthcare sector. This study addresses this empirical gap by investigating these mediation pathways, thereby contributing novel insights to the existing body of literature. In line with this conceptual framework, the following hypotheses were formulated:

### H1

Green competencies are positively related to nurses’ sustainable practices.

### H2

Green competencies are positively associated with environmental awareness.

### H3

Green competencies are positively associated with climate change awareness.

### H4

Environmental awareness mediates the association between green competencies and nurses’ sustainable practices.

### H5

Sustainable practices are positively associated with climate change awareness.

### H6

Climate change awareness mediates the relationship between green competencies and nurses’ sustainable practices.

### H7

Sustainable practices are positively associated with nurses’ environmental awareness.

### H8

Environmental awareness and climate change awareness serially mediate the association between green competencies and nurses’ sustainable practices.

**Fig. 1:**
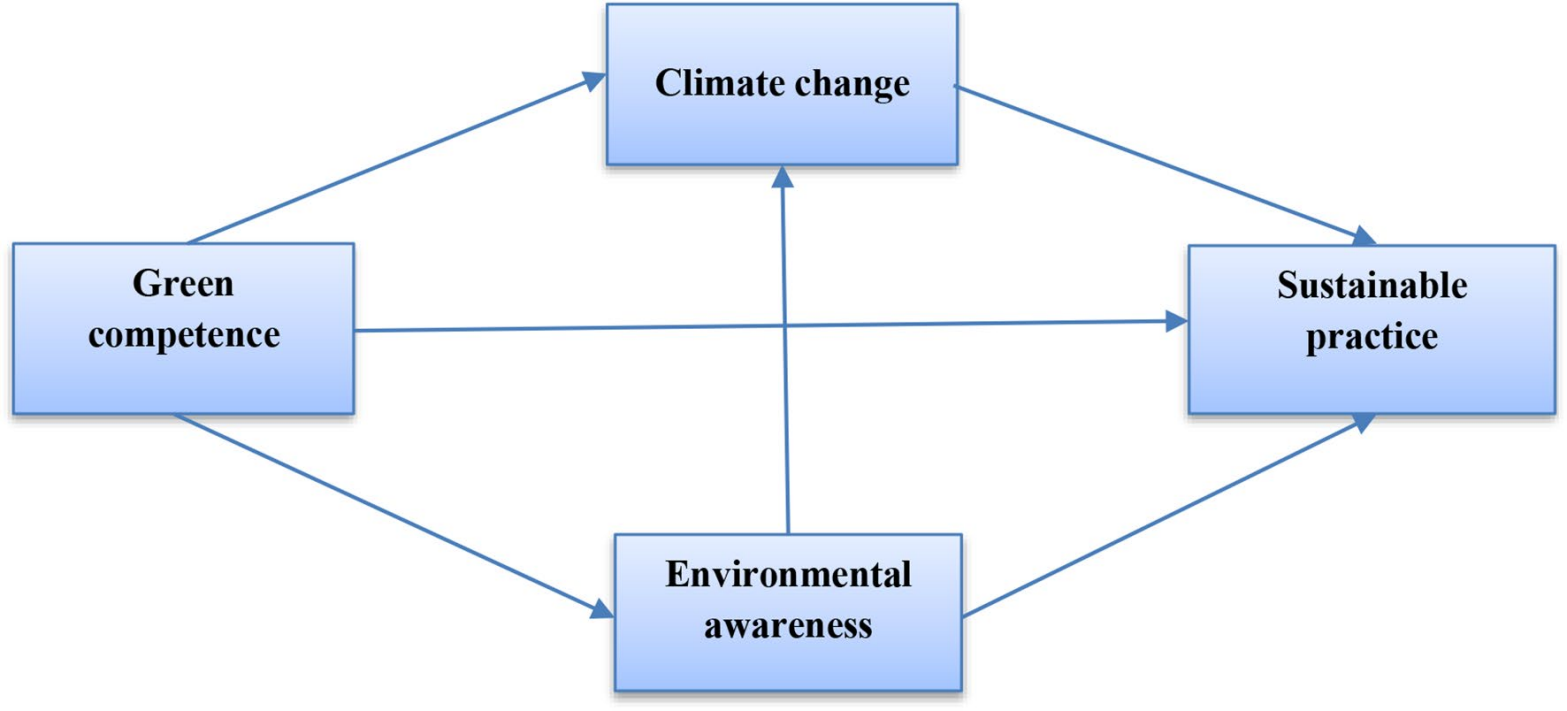
Conceptual model

Taking into account the eight hypotheses outlined above, the conceptual research model is presented as follows (Fig. 1):

## Methods

Aim of the study: This study aimed to investigate the relationships among green competencies, nurses’ sustainable practices, environmental awareness, and perceptions of climate change. Additionally, it introduces a mediation model examining environmental awareness and climate change as mediating variables in the relationship between green competencies and sustainable nursing practices at Al Ahrar Teaching Hospital, Zagazig, Egypt.

### Setting and study design

A descriptive correlational design was employed to conduct this study. The research was carried out at Al-Ahrar Teaching Hospital, a prominent 450-bed facility located in Zagazig City, Sharkia Governorate, Egypt. Established in 2007, the hospital operates under the General Authority for Hospitals and Educational Institutes and serves as a central healthcare provider in the region. Its extensive range of services including inpatient care, outpatient clinics, emergency services, intensive care units (ICU and NICU), and specialized departments caters to a diverse patient population from both urban and rural areas. In 2024, the hospital reported high patient volumes, including over 118,000 cases in the emergency department, more than 250,000 outpatient visits, 5000 GIT endoscopy, and approximately 115,000 surgical procedures, encompassing 146 open-heart surgeries and 213 cardiac catheterizations. The hospital’s significant role in the community, combined with its diverse patient demographics and comprehensive healthcare services, makes it an ideal setting for examining the relationships between green competence, environmental awareness, climate change perception, and sustainable practices among nurses.

### Participants

A sample of 230 nurses from diverse departments was recruited for the study. The inclusion criteria for the study required nurses to be currently employed at Al-Ahrar Teaching Hospital, aged between 30 and 40 years, and possess at least a diploma in nursing, with many higher qualifications such as a bachelor’s or postgraduate degree in nursing. Participants were selected based on their active involvement in clinical practice across various departments, including emergency, intensive care, and surgical units. The exclusion criteria included nurses on extended leave, those with less than six months of employment at the hospital, and nurses working in administrative or non-clinical roles, as their experience would not provide insight into the specific practices examined in the study.

The G*Power (3.1.9.7) program was used to determine the required sample size, employing calculations based on F tests in the context of linear multiple regression with a fixed model and the R² deviation from zero. This a priori analysis computed the necessary sample size. A simple random sampling technique was employed using a computer-generated random number list to select participants from a complete registry of eligible nurses provided by the hospital’s human resources department. The sample consisted of nurses aged between < 30 and > 40 years, with varying educational backgrounds, including Diploma Nurses, Technical Institute of Nursing graduates, Bachelor’s degree holders in nursing, and those with postgraduate nursing education. These nurses are currently employed across different hospital departments.

#### Instruments for data collection

Five instruments were employed to gather data for this study, which comprised: -.

#### Tool one: nurses’ sociodemographic characteristics questionnaire

Created by researchers in their native language (Arabic), this section provides a comprehensive list of the various personal characteristics of nurses, including age, gender, education level, job position, residence, marital status, years of experience, monthly income, department in the hospital. Each item was carefully chosen to paint a complete picture of the diverse demographic and professional attributes of the participating nurses. This meticulous approach to data collection was intended to ensure that the information gathered was both high-quality and relevant, significantly enhancing the understanding of the research context and facilitating more rigorous analyses in future studies.

2nd instrument: The Green Competence Scale (GCS), developed by Cabral and Lochan Dhar (2019) [23]. It was adopted and modified to be suitable for assessing green competencies among nurses. The scale consists of 31 items grouped into six dimensions: green knowledge (3 items), green skills (3 items), green abilities (4 items), green attitude (7 items), green behavior (10 items), and green awareness (4 items). The internal consistency of the scale was assessed with Cronbach’s alpha of 0.813, and validity was confirmed through expert review and pilot testing. To ensure content validity, the scale was reviewed by a panel of three experts one in nursing administration, one in Community health nursing and the last one in green practices to verify that the items were relevant and appropriate for the nursing context. Each item on the scale was rated on a five-point Likert scale, ranging from 1 = strongly disagree to 5 = strongly agree, with higher scores indicating a greater level of green competencies among nurses.

The Cronbach’s alpha values for each dimension were as follows: green knowledge (0.862), green skills (0.831), green abilities (0.879), green attitude (0.885), green behavior (0.883), and green awareness (0.783). The overall Cronbach’s alpha for this study was 0.813, demonstrating strong internal consistency. Additionally, confirmatory factor analysis (CFA) was conducted on the translated version of the scale to assess its factor structure, with goodness-of-fit indices indicating a satisfactory model fit (Comparative Fit Index [CFI] = 0.94, Tucker-Lewis Index [TLI] = 0.92, Root Mean Square Error of Approximation [RMSEA] = 0.06), confirming the scale’s validity and robustness for use in this context.

3rd instrument: The Nurses’ Perception Toward Climate Change Scale was developed by the researcher for the current study, based on an extensive literature review including the works of Buriro et al. (2018), La Torre et al. (2020), and Anåker, Spante, & Elf (2021) [6, 24, 25]. The scale consists of 16 items, and was designed to assess staff nurses’ perceptions of climate change. The scale was created in Arabic, the participants’ native language, ensuring cultural and contextual relevance for the target population. The internal consistency reliability of the scale was assessed with a Cronbach’s alpha of 0.913, demonstrating strong reliability. Each item on the scale was rated using a three-point Likert scale, ranging from 1 = strongly disagree to 3 = strongly agree, with higher scores indicating a stronger perception of climate change among nurses. To confirm the scale’s validity, content validity was evaluated by a panel of three expert groups, consisting of one expert in nursing administration, one expert in community health nursing, and one expert in green practices. These experts reviewed the scale to ensure its relevance and appropriateness for the nursing context. The expertise of the individuals involved in the content validity process contributed to a thorough assessment of the scale’s cultural and contextual appropriateness. Additionally, confirmatory factor analysis (CFA) was conducted on the Arabic version of the scale to assess its factor structure and ensure the validity of the tool. Goodness-of-fit indices showed a satisfactory model fit (Comparative Fit Index [CFI] = 0.95, Tucker-Lewis Index [TLI] = 0.93, Root Mean Square Error of Approximation [RMSEA] = 0.05), confirming the scale’s validity and robustness for use in this context.

4th instrument: Nurses Environmental awareness tool (NEAT-es): The Environmental Awareness Scale for Nurses, originally developed by Luque-Alcaraz (2022) [26]. It was translated and adapted into Arabic for this study to ensure linguistic and cultural suitability for Arabic-speaking participants. This 31-item instrument comprises three subscales: the Nurse Awareness Scale (NAS) with 11 items, the Nurse Professional Ecological Behaviors Scale (NPEB) with 9 items, and the Personal Ecological Behaviors Scale (PEB) with 11 items. To ensure a linguistically and conceptually accurate translation, the scale underwent a rigorous forward–backward translation process. First, two independent bilingual nursing experts translated the original English version into Arabic. Then, a separate pair of bilingual experts who were blinded to the original version performed the back-translation into English. Discrepancies were discussed and resolved by consensus to maintain semantic and conceptual equivalence. Content validity was assessed by a panel of experts in nursing administration, Community health nursing and green practices, ensuring relevance and appropriateness for the local context. Additionally, confirmatory factor analysis (CFA) was conducted to evaluate the factor structure of the Arabic version, with goodness-of-fit indices showing a satisfactory model fit (Comparative Fit Index [CFI] = 0.93, Tucker-Lewis Index [TLI] = 0.92, Root Mean Square Error of Approximation [RMSEA] = 0.05), confirming the scale’s structural validity. All items were positively worded, with responses rated on a 5-point Likert scale from 1 (strongly disagree) to 5 (strongly agree), where higher scores reflected stronger environmental awareness and ecological behavior. The Arabic version demonstrated strong internal consistency reliability, with a Cronbach’s alpha of 0.938.

5th instrument: The Sustainable Development Behavior Scale: adapted from Dumitru et al. (2015) [27] and modified by the researcher following a thorough literature review, was designed to assess nurses’ sustainable health behavior levels in the workplace. This 17-item scale is organized into three domains: environmental (5 items), social (6 items), and economic (6 items). Each item is rated on a five-point Likert scale, from 1 (strongly disagree) to 5 (strongly agree), with higher scores indicating greater engagement in sustainable development behaviors among nurses. To ensure the tool’s validity, multiple assessments were conducted. Content validity was established through expert review, with specialists in sustainable development and nursing confirming that the scale comprehensively reflect sustainable behaviors across environmental, social, and economic dimensions relevant to nursing. Construct validity was evaluated using confirmatory factor analysis (CFA), which confirmed the expected three-factor structure with satisfactory goodness-of-fit indices (Comparative Fit Index [CFI] = 0.92, Tucker-Lewis Index [TLI] = 0.91, Root Mean Square Error of Approximation [RMSEA] = 0.06). Additionally, criterion validity was examined by correlating the scale with established measures of sustainable behavior, showing strong positive correlations that reinforce the tool’s accuracy in assessing sustainable health behaviors. The scale’s high internal consistency reliability (Cronbach’s alpha = 0.932) further supports its robustness and suitability for evaluating sustainable development behaviors among nurses.

### Procedure

Before the commencement of the primary research study, a preliminary investigation referred to as a pilot study was conducted on a randomly selected cohort of 23 nurses, constituting 10% of the total nurses under examination. The primary objectives of this pilot study were to assess the clarity, applicability, and practicality of the study instruments, gauge the time required for participants to complete the instruments, and identify any potential challenges anticipated during data collection. The outcomes of the pilot study indicated that no modifications were deemed necessary; the instruments exhibited clarity and vibrancy. Notably, participants involved in the pilot study were deliberately excluded from the overall sample of the research to ensure result consistency.

During the data collection phase, which spanned approximately sixteen weeks, we met the participant nurses and conducted face-to-face interviews with them in various departments in the hospital; the research objectives were explained to the nurses, and their ability to participate was assessed. Upon obtaining their written consent, we scheduled and conducted in-person interviews at times suitable for the nurses. Each interview session lasted between 30 and 45 min, which included the time required to complete all research instruments. The researchers followed a structured questionnaire to gather responses, ensuring consistency and accuracy in data collection. Responses were documented in real time by the interviewers using a paper-based format that was immediately checked for completeness and clarity at the end of each session.

The survey was comprised of two parts: the first part was the informed consent, which explained the study’s aim, confidentiality guarantees, and the ability to withdraw at any time. This informed consent form was included in one document with the research tool. Participants had to agree to the terms before continuing with the questionnaire, ensuring they fully understood the study before proceeding. The second part included the research instrument. To minimize interviewer bias, the research team received standardized training on administering the tools objectively, and a neutral tone was maintained during all interactions. Interviewers avoided leading questions, and responses were recorded verbatim to ensure authenticity and accuracy. In this phase, efforts were made to create a comfortable and supportive environment, encouraging honest and comprehensive responses from the participants.

### Ethical consideration

Ethical approval for this study was obtained from the Research Ethics Committee (REC) at the faculty of Nursing, Zagazig University, Egypt (Approval No. ID/ZU.Nur.R.E.C.#140). The study’s objectives were thoroughly explained to participants, emphasizing that all collected data would be used solely for research purposes. Each participant was informed of their right to refuse participation or to withdraw at any point before completing the study materials without any adverse consequences. Written informed consent was obtained from all nurses who chose to participate. To ensure confidentiality and data security all responses were anonymized, and data access was restricted exclusively to the research team. Data was secured and handled in strict accordance with the ethical standards outlined by the Declaration of Helsinki and its subsequent amendments, thereby safeguarding participants’ rights and well-being throughout the study.

### Data analysis

Data analysis was performed using SPSS 26.0 (IBM Inc., Chicago, IL, USA) to examine the survey responses from the *230* recruited nurses. Descriptive statistics, such as frequencies (percentages) and mean ± standard deviations (SD), were utilized to summarize both the general characteristics of the participants and the scores obtained on various scales. Correlations between environmental awareness, climate change and green competence among nurses were evaluated using Pearson’s correlation analysis. Additionally, the AMOS software (version 26.0; IBM SPSS Amos) was employed to develop and test the hypothesized path model. Structural Equation Modeling (SEM) using AMOS allowed for the evaluation of direct and indirect relationships among study variables, providing insights into the mediating role of environmental awareness and climate change in shaping green competence.

## Results

Table 1 shows that that over half of the participant nurses (57.3%) were under 30 years old, with 59.6% being female and 57% married. Additionally, 61.4% of participants held qualifications from a Technical Nursing Institution, and 58.2% had more than 10 years of experience in nursing. Statistically significant differences were observed in study variables based on participants’ age, years of experience, and marital status (*p* < 0.01). Insert Table 1.

**Table 1:** Participants demographics and differences in study variables (*N* = 230 )

Characteristic	Category	No. (%)	Green competence		Climate change		Environmental awareness		Sustainable practice	
			M (SD)	t/f (p)	M (SD)	t/f (p)	M (SD)	t/f (p)	M (SD)	t/f (p)
Gender	Male	93 (40.4)	126.45 (11.86)	F = 2.55 (0.112)	65.13 (5.7)	F = 2.397 (0.123)	111.24 (15.07)	F = 72.04 (0.000)	67.24 (10.8)	F = 8.433 (0.004)
	Female	137 (59.6)	128.53 (7.90)		67.17 (11.84)		128.04 (14.5)		71.17 (9.6)	
Age	less than 30	132 (57.3)	126.17 (8.3)	F = 29.261 (0.000)	69.39 (5.4)	F = 283.700 (0.000)	111.2366	F = 38.349 (0.000)	69.35 (10.0)	F = 40.700 (0.000)
	30–40	70 (30.4)	133.64 (10.8)		69.56 (6.03)		128.59 (11.73)		74.99 (8.22)	
	More than 40	28 (12.3)	120.0 (0.0)		44.0 (0.0)		100.0 (0.0)		57.18 (0.39)	
Qualification	Technical institution	141 (61.4)	127.89 (9.51)	F = 0.146 (0.703)	65.69 (11.2)	F = 1.634 (0.202)	121.58 (16.3)	F = 0.130 (0.719)	67.87 (11.2)	F = 10.554 (0.001)
	B.S.C	89 (38.6)	127.38 (10.11)		67.4 (7.2)		120.74 (17.8)		72.29 (7.8)	
Experience	less than 10 years	134 (58.2)	126.2 (8.3)	F = 5.872 (0.003)	69.38 (5.4)	F = 71.948 (0.000)	121.97 (17.19)	F = 12.097 (0.000)	69.49 (10.0)	F = 12.893 (0.000)
	10–20	56 (24.3)	131.39 (10.53)		68.62 (6.36)		126.98 (12.6)		73.98 (8.9)	
	More than 20 years	40 (17.3)	127.5 (11.6)		53.0 (13.9)		110.80 (16.71)		63.72 (10.13)	
Marital status	Single	99 (43)	129.63 (9.4)	F = 7.057 (0.008)	70.16 (5.11)	F = 29.162 (0.000)	126.88 (14.7)	F = 21.060 (0.000)	71.84 (7.9)	F = 8.689 (0.004)
	Married	131 (57)	126.23 (9.8)		63.47 (11.5)		116.99 (17.2)		67.88 (11.4)	

(M) Mean. (SD) Standard deviation. (a) (F) One-way analysis of variance. (b) *t*-test for the independent group. * *(p) Significance level* < 0.01

Table 2 illustrates strong correlation between green competence and sustainable practice (*r* = 0.724, *p* < 0.01), environmental awareness (*r* = 0.662, *p* < 0.01), and climate change (*r* = 0.551, *p* < 0.01). These results suggest that nurses with higher levels of green competence are more likely to engage in sustainable practices, exhibit environmental awareness, and adopt climate change strategies in their workplace. Additionally, sustainable practice showed positive correlations with climate change (*r* = 0.632, *p* < 0.01) and environmental awareness (*r* = 0.690, *p* < 0.01). This implies that nurses who are more attuned to their work environment and adapt to climate conditions tend to enhance their sustainable practices. Furthermore, climate change was positively correlated with environmental awareness (*r* = 0.826, *p* < 0.01), indicating that as nurses develop greater awareness of environmental issues they are more likely to recognize and address climate change in their professional practices. Insert Table 2.

**Table 2:** Correlations among the study variables (*N* = 230)

Variables	Mean (SD)	α	Green Competence	Climate Change	Environmental Awareness	Sustainable Practice
Green Competence	127.7 (9.7)	0.813	1	0.551**	0.662**	0.724**
Climate Change	66.35 (9.9)	0.913	0.551**	1	0.826**	0.632**
Environmental Awareness	121.2 (16.9)	0.938	0.662**	0.826**	1	0.690**
Sustainable Practice	69.6 (10.3)	0.932	0.724**	0.632**	0.690**	1

Correlation.* Correlation is significant at the 0.05 level (2-tailed). **Correlation is significant at the 0.01 level (2-tailed)

**Fig. 2:**
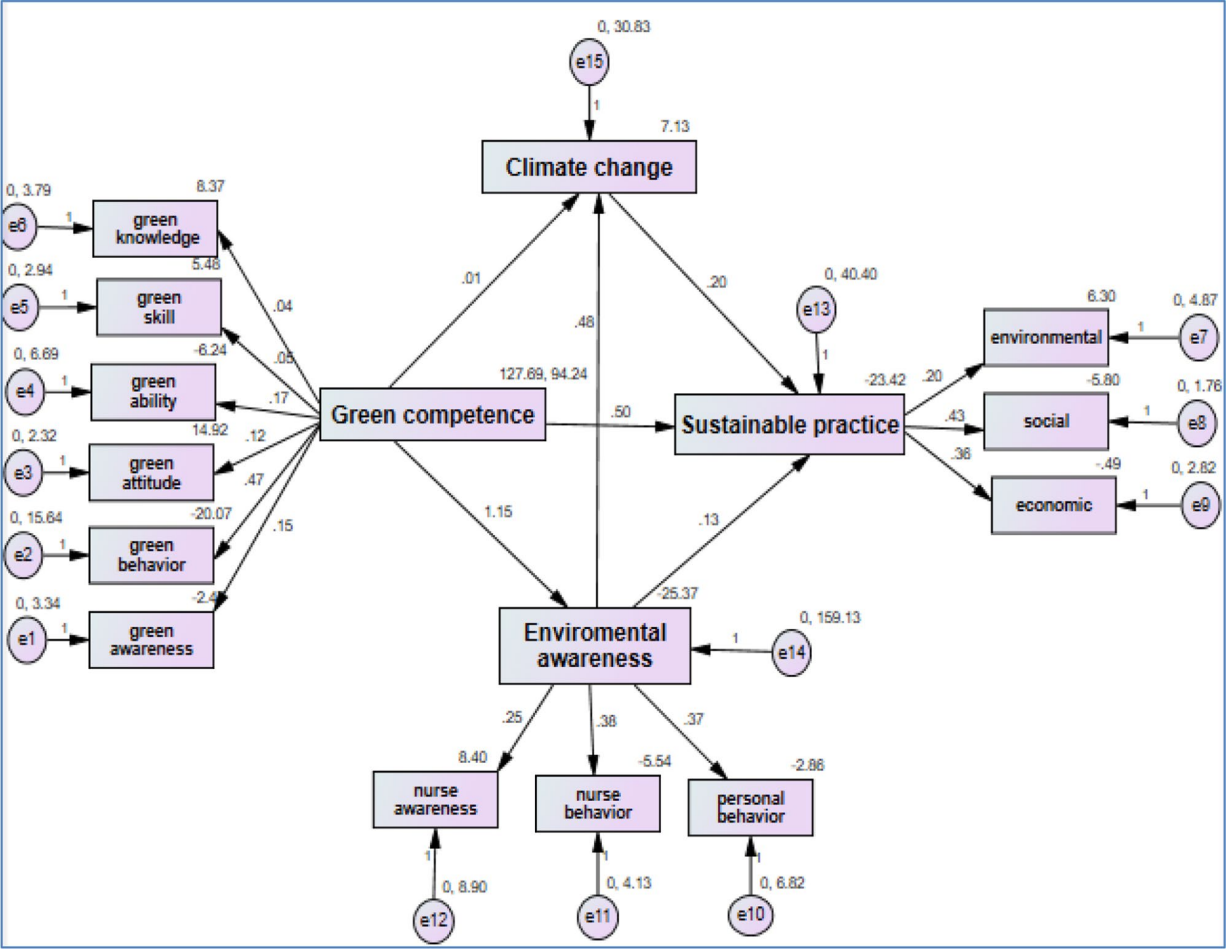
The mediating effect of environmental awareness and climate change on the relationship between green competencies and nurses sustainable practice (*N* = 230)

Table 3 illustrates the direct and indirect effects. Also, Fig. 2 pictures the mediating effect of environmental awareness and climate change on the relationship between green competence and sustainable practice. As displayed green competence had significant direct effect on environmental awarenss (β = 1.148 *p* < 0. 01), climate change (β = 0.872, *p* < 0. 01) and sustainable practice among nurses (β = 0.944, *p* < 0. 01). Likewise, environmental awareness had significant direct effect on climate change (β = 0.480, *p* < 0. 01) and sustainable practice (β = 0.135, *p* < 0. 01). As well, climate change had a significant direct effect on sustainable practice (β = 0.196, *p* < 0. 01). Isert Table 3.

**Table 3:** Tests of direct and indirect effects (*N* = 230)

Effects	β	*P*	*Percentile 95% CI* *Lower/Upper*
Direct effects:			
Green competence to Environmental awareness	1.148	0.000	1.037/1.264
Green competence to climate change	0. 872	0.008	0.067/0.081
Green competence to sustainable practice	0.499	0.000	0.381/0.620
Environmental awareness to climate change	0.480	0.000	0.421/0.548
Environmental awareness to sustainable practice	0.135	0.006	0.041/0.244
climate change to sustainable practice	0.196	0.010	0.060/0.356
**Indirect effects:**			
Green competence to sustainable practice via Environmental awareness	0.094	0.000	0.029/0.172
Green competence to sustainable practice via climate change	0.551	0.000	0.465/0.652
Green competence to sustainable practice via Environmental awareness and climate change	0.264	0.000	0.187/0.341

* *(p) Significance level* ≤ 0.05. **(p) Significance level < 0.001

Correspondingly, there was significant indirect effect of environmental awareness on the relationship between green competence and sustainable practice (β = 0.094, *p* = 0.000, 95% CI 0.029/ 0. 172), representing that the relationship between green competencies and sustainable practice among staff nurses was partially mediated by environmental awareness. Climate change also had a significant indirect effect on the relationship between green competence and sustainable practice (β = 0.551, *p* < 0.001, 95% CI -0.465/0.652), demonstrating that climate change partially mediated the relationship between green competence and sustainable practice among staff nurses. Furthermore, environmental awareness and Climate change were found to have a significant indirect effect on the relationship between green competence and sustainable practice (β = 0.264, *p* < 0.001, 95% CI 0.187/0.341) signifying that these two factors acted as a partial mediator of the relationship between green competence and sustainable practice among staff nurses. Isert Figure 2.

## Discussion

Sustainability of biological, social, and economic systems is crucial for protecting our common future and preserving the balance between nature and humans [28]. Environmental concerns should be adopted by all units of society and sustainability awareness should be adapted to all processes through optimum technologies both in daily life and in business management [29]. All of these require green competencies that help in managing climate changes. Thus the purpose of this study is to investigate the mediating effect of environmental awareness and climate change on the relation ship between green competencies and sustainable practice.

The result of our study found that green competencies have a strong and significant direct effect on nurses sustainable practice. These results are in the same line with finding of the study preformed by Ozilhan and colleagues, (2024) [30]. Also the findings by Abdelkareem and colleagues, (2024) agreed with these findings and reported that green competencies has positive effect on sustainable practice [31]. The results of the present study reported that green competencies also has a robust direct effect on environmental awareness indicating that high green competencies levels directly enhance environmental awareness to their jobs. These results were supported by Trisnawati and Muafi, (2022) who showed that the direct effect of green competencies and environmental awareness is significant [32]. Also Mansour, (2023) revealed that there is a noteworthy direct correlation between green competencies and environmental awareness [33].

Furthermore, green competencies have a strong and significant direct effect on climate change. Green competencies actively motivate nurses to engage in eco-friendly processes and green activities by providing the necessary information for them to face and manage climate change and positively promote sustainable performance. This result in the same vein with the findings by Shaban and colleagues, (2024) [34]. Also, These findings were supported by Younis & Hussain (2023) who found that green competencies and climate change is significantly and positively associated [35].

Additionally, environmental awareness has a significant effect on sustainable practice and climate change [36]. Our study demonstrated that environmental awareness significantly enhances sustainable practices and supports climate change mitigation, underscoring its critical role in promoting sustainability among nurses. Cultivating employees’ environmental awareness and fostering sustainability consciousness are essential for encouraging the adoption of sustainable practices. Moreover, individuals’ environmental consciousness and awareness of ecological issues play a pivotal role in developing green competencies and productivity, driving them to prioritize environmental concerns. These findings align with those of Dönmez and Yardımcı (2024), who reported that environmental awareness has a significant and positive impact on nurses’ sustainable practices [37]. Furthermore, this result aligns with the findings of Wu and colleagues (2024) and Neruja and Arulrajah (2021), which identified a positive correlation between environmental awareness and sustainable practices [38, 39].

Regarding the effects of climate change, the current study revealed that these effects had a significant positive direct effect associated with sustainable practices. Environmental awareness has played a crucial role in mitigating the effect of climate change and can assist nurses in addressing challenges related to unsustainable clinical practices. This result agreed with Mekawy (2023) and Anaker and colleagues, (2021) who found that climate change and sustainability practice in nursing are significantly associated [6, 40]. While contradicted with the finding of Hassan et al. (2022) who revealed that there was a significant and negative effect between the implementation of environmental and the challenges of implementing sustainable practices [41].

Our study revealed that both environmental awareness and climate change had a significant indirect effect on the relationship between green competencies and sustainable practices. Similarly, Wu and colleagues (2024) and Kousar and colleagues (2022) reported that environmental awareness significantly influenced the relationship between green competencies and sustainable practices [38, 42]. Furthermore, this result aligns with the findings of Younis and Hussain (2023), who reported that climate change had a significant indirect effect on the relationship between green competencies and sustainable practices [35]. In contrast to these results, Trisnawati and Muafi (2022) and Ahmed and colleagues (2021) reported that employee wellbeing mediates the relationship between green competencies and sustainable practices [32, 43]. Additionally, a study by Yafi and colleagues (2021) found that motivation had a significant indirect effect on the relationship between green competencies and sustainable practices [44].

## Conclusion

This study highlights green competence as a key factor in promoting sustainable practices among nurses, with environmental awareness and climate change serving as significant mediators. Findings show that higher levels of green competence strongly correlate with increased engagement in sustainable practices and environmental awareness. Addressing these dynamics through targeted training, increased environmental knowledge, and climate change education can enhance sustainable practices and improve healthcare outcomes, leading to a more environmentally conscious nursing workforce.

### Implication

This study highlights important implications for nursing management and the broader healthcare sector. It emphasizes the need for investing in the development of green competencies among nurses, which involves offering training and education on sustainability practices, environmental awareness, and climate change. By equipping nurses with these competencies, healthcare organizations can encourage active participation in sustainability initiatives, thus enhancing environmental responsibility and overall organizational performance. Nursing managers should prioritize integrating sustainability practices across various departments and processes, while efficiently allocating resources to projects and initiatives that foster sustainable outcomes.

### Limitation of the study

While this study offers valuable insights into how environmental awareness and climate change mediate nurses’ green competence and sustainable practices, several limitations warrant consideration. The cross-sectional design limits causal inference, and the single-site sampling from an Egyptian teaching hospital may constrain the generalizability of findings. Although self-reported measures provided practical access to participants’ perspectives, they may be subject to response bias. Additionally, contextual influences such as institutional policies or culturally specific interpretations of adapted instruments may have subtly shaped responses. To enhance future research, longitudinal and multi-center studies are recommended to establish causality and broaden applicability. Combining objective assessments (e.g., observational audits) with self-reports could improve measurement accuracy. Further, exploring organizational factors such as leadership support and resource availability, as well as implementing interventional studies on green competence training, could yield deeper insights. Qualitative inquiries into nurses’ perceived barriers to sustainability may also help design scalable and culturally attuned strategies for the healthcare sector.

## Data Availability

The corresponding author can provide the datasets used and/or analyzed for this study upon reasonable request.

## Abbreviations

COPD: Chronic Obstructive Pulmonary Disease
REC: Research Ethics Committee
CFI: Comparative Fit Index
CFA: Confirmatory Factor Analysis
GCS: Green Competence Scale
RMSEA: Root Mean Square Error of Approximation
NEAT: Nurses Environmental Awareness Tool
NAS: Nurse Awareness Scale
NPEBS: Nurse Professional Ecological Behaviors Scale
PEB: Personal Ecological Behaviors
